# Strategies and Structure Feature of the Aboveground and Belowground Microbial Community Respond to Drought in Wild Rice (*Oryza longistaminata*)

**DOI:** 10.1186/s12284-021-00522-8

**Published:** 2021-09-08

**Authors:** Jian Xie, Xiaoqing Wang, Jiawang Xu, Hongwei Xie, Yaohui Cai, Yizheng Liu, Xia Ding

**Affiliations:** 1 School of Life Sciences, Nanchang University, Nanchang, 330031 Jiangxi China; 2 Jiangxi Super-Rice Research and Development Center, Jiangxi Academy of Agricultural Sciences, Nanchang, 330200 Jiangxi China

**Keywords:** Plant microbiome, Wild rice, Drought, *Oryza longistaminata*, Microbial community assembly, Host microbial ecology

## Abstract

**Background:**

Drought is global environmental stress that limits crop yields. Plant-associated microbiomes play a crucial role in determining plant fitness in response to drought, yet the fundamental mechanisms for maintaining microbial community stability under drought disturbances in wild rice are poorly understood. We make explicit comparisons of leaf, stem, root and rhizosphere microbiomes from the drought-tolerant wild rice (*Oryza longistaminata*) in response to drought stress.

**Results:**

We find that the response of the wild rice microbiome to drought was divided into aboveground–underground patterns. Drought reduced the leaf and stem microbial community diversity and networks stability, but not that of the roots and rhizospheres. Contrary to the aboveground microbial networks, the drought-negative response taxa exhibited much closer interconnections than the drought-positive response taxa and were the dominant network hubs of belowground co-occurrence networks, which may contribute to the stability of the belowground network. Notably, drought induces enrichment of Actinobacteria in belowground compartments, but not the aboveground compartment. Additionally, the rhizosphere microbiome exhibited a higher proportion of generalists and broader habitat niche breadth than the microbiome at other compartments, and drought enhanced the proportion of specialists in all compartments. Null model analysis revealed that both the aboveground and belowground-community were governed primarily by the stochastic assembly process, moreover, drought decreased ‘dispersal limitation’, and enhanced ‘drift’.

**Conclusions:**

Our results provide new insight into the different strategies and assembly mechanisms of the above and belowground microbial community in response to drought, including enrichment of taxonomic groups, and highlight the important role of the stochastic assembly process in shaping microbial community under drought stress.

**Supplementary Information:**

The online version contains supplementary material available at 10.1186/s12284-021-00522-8.

## Background

Drastic climate change and increased water scarcity are challenges to global crop production (de Vries et al. 2018). Drought is one of the most important factors influencing rice production and can lead to a 25% decrease in rice yield (Zhang et al. 2018). During rice domestication, 50–60% of alleles were lost from the nearest wild relatives of cultivated rice (*Oryza sativa*) (Melaku et al. 2013; Wambugu et al. 2013). Cultivated rice (*O. sativa*) is more sensitive to drought than wild rice (Zhang et al. 2018; Daryanto et al. 2017). Wild rice may serve as a source of superior drought tolerance alleles for cultivated rice. There is an urgent need to explore wild rice drought stress resistance mechanisms, which can then be used to improve cultivated rice productivity under unfavorable drought stresses conditions given the limited global land resources.

Understanding the fundamental mechanisms for maintaining and generating species diversity in response to drought distribution is pivotal to determine the links between community stability and ecosystem function (Jiao and Lu 2020; Meyer et al. 2018; Hanson et al. 2012). Drought not only decreases the water available, nutrient availability, N, and P uptake to microbial communities and plants but also changes the microbial diversity and community stability (Hartman and Tringe 2019). The microbiome can exhibit remarkable stability in response to environmental disturbances in natural ecosystems, mainly due to their extreme physiological tolerance, high metabolic flexibility, large population size, widespread dispersal and rapid growth (Jiao et al. 2019). The assembly mechanisms of crop microbiome on drought disturbances are still unclear. Microbial communities control many biogeochemical processes in crop-ecosystems (Martiny et al. 2013). Given microbial importance to crop-ecosystem functioning and services, it is imperative that microbial communities are maintained in a stable state following drought disturbances. Understanding the biodiversity-stability relationship and maintaining microbial diversity in drought-tolerant wild rice under drought stress is critical in efforts to improve rice yields under increasingly frequent global droughts.

A key issue in microbial ecology is quantifying the relative importance of stochastic and deterministic processes in mediating microbial community assembly (Zhou et al. 2014; Zhou and Ning 2017; Dini-Andreote et al. 2015; Costello et al. 2012). To date, extensive evidence suggests that microbiota community establishment around plants is not random but is rather controlled by specific assembly rules (Vorholt 2012; Bulgarelli et al. 2013; Reinhold-Hurek et al. 2015). Although the mechanisms shaping microbial community structure and stability have been intensively examined, those controlling the ecological processes of crop systems in response to environmental perturbations remain unclear. To understand the mechanisms and factors giving structure to natural and agricultural microbial communities, studies have emphasized two types of processes: stochastic and deterministic (Gao et al. 2020). Both stochastic and deterministic components are embedded in four ecological processes, including selection, dispersal, diversification, and drift (Zhou and Ning 2017). The balance between stochastic and deterministic assembly processes is mediated by environmental factors (Jiao and Lu 2020). Several recent studies have provided an excellent overview of the ecological processes controlling microbial community structure and biogeographic patterns in general (Dini-Andreote et al. 2015; Hanson et al. 2012; Nemergut et al. 2014; Costello et al. 2012; Zhou et al. 2014; Stegen et al. 2013).

Red rice, *Oryza longistaminata*, is perennial wild rice with strong rhizomes (Wambugu et al. 2015). *O. longistaminata* contains various agronomically valuable traits that could be used in rice improvement programs, including drought tolerance, long anthers, large biomass in poor soils, high nitrogen use efficiency, and resistance to insect pests and disease (Yang et al. 2010). Along with its useful traits, *O. longistaminata* possesses an AA genome type (2n = 24) and is closely related to cultivated rice (*O. sativa*), which makes it a good candidate for rice breeding (He et al. 2014). In light of growing concerns over the threat of water stress to agricultural ecosystems, increased emphasis has been placed on a mechanistic understanding of how drought stress conditions influence the composition and functioning of the plant-association microbiome and the ultimate consequences for plant health (de Vries et al. 2020).

By exploiting this unique agronomically trait in wild rice (*O. longistaminata*), we aimed to disentangle the relationships between plant microbiome and host systems and to address the fundamental mechanisms of microbiota communities’ stability and recruitment strategies in wild rice under drought conditions. However, it is rare to make explicit comparisons of leaf, stem, root and rhizosphere microbiomes from the same plant, and it is largely unknown whether these microbiomes will change on the same spatial scale in response to similar drought stress. Here, we focus on the following questions: First, comprehensively analyze how drought impacts the structure feature of the leaf, stem, root and rhizosphere microbiome in drought-tolerant wild rice? Second, which deterministic and/or stochastic processes determined the microbial community assembly in response to drought perturbations? To answer these questions, we compared the microbial community structures, network structures, and assembly processes within the leaf, stem, root and rhizosphere microbiome of *O. longistaminata* in response to drought stress. The results from our experiments provide insight into adaptive plant responses to global change by identifying changes in the plant microbiome.

## Methods

### Sample Collection and Processing

The site of the rice experiment is in Nanchang city in China. Soil from the rice field in Nanchang city (28° 40′ 04″ N, 115° 49′ 31″ E) was collected using shovels. Then all soil was mixed well to homogenize the soil. For soil texture analyses, stones and visible plant residues were removed from air-dried soil samples before being passed through a 2 mm sieve. Soil texture was measured by the hydrometer method (Ashworth et al. 2001). Soil is typically classified as sandy clay loam (Additional file 7: Table S1). The soil was placed into 680 × 520 × 385 mm pots. Seeds of *Oryza longistaminata* were grown in the pots in May 2017. Each treatment had 6 pots, giving a total of 12 pots. Each pot contained 6 rice plants. The wild rice was watered with tap water. All weeds were manually removed from the pots when identified. Drought stress was imposed on 3-month-old plants by ceasing irrigation and letting the soils progressively dry down. The drought treatment lasted for 2 months.

At the end of October, samples were collected from four compartments (the rhizosphere, leaf, stem, and root) and bulk soil. Four plant and rhizosphere soil samples were collected from each pot, and four plant samples were pooled into one sample, four rhizosphere soil were pooled into two samples, giving a total of 60 plant and rhizosphere samples and 6 bulk soil samples. The plant was removed from each pot. Loosely bound soil was shaken off from the plant roots, and tightly bound soil samples (rhizosphere soil) were collected by brushing off the soil that tightly adhered to the roots. The rhizomes were carefully removed from the root. All samples were packed into polyethylene bags and shipped on ice packs (4 °C) to the laboratory. Ethanol-sodium hypochlorite was used for surface sterilization (Tamai and Ma 2003; Sun et al. 2008; Zhang et al. 2013). Fragments of the roots, stems, and leaves were washed with sterile water and separated. Then the rice samples were ultrasound-treated. All the samples were washed successively in 70% ethanol for 1 min and 0.3% sodium hypochlorite with 0.01% Tween 20 for 15 min to further clean the surfaces of the living microbiome, and the samples were subsequently washed three times in sterile water. Finally, the sterile filter paper was used to absorb any extra moisture. The water used for the final wash was spread on the LB and PDA plates to verify examine the surface sterilization effect. Each sample was stored at − 80 °C for DNA extraction.

### Soil Water Content Analyses

Five soil samples were collected from 0 to 20 cm using soil shovels per pot, five soil samples were pooled into one sample, giving a total of 6 soil samples per treatment. The water content of the soil samples was measured gravimetrically by oven-drying 5 g of freshly collected soil at 105 °C for 24–48 h until the soil reached a constant weight, the water content was calculated as water/(water + soil) (w/w).

### Photosynthetic Parameter Analysis

To evaluate the effect of the drought treatments on *Oryza longistaminata* performance, we measured photosynthetic parameters, net photosynthetic rate (Pn), stomatal conductance (Cs), intercellular CO_2_ concentration (Ci), transpiration rate (Tr), stomatal limitation value (Ls), and water use efficiency (WUE). The gas exchange parameter was measured through a portable photosynthesis gas exchange system (IRGA, Model LI-6400XT, Li-Cor, Lincoln, Nebraska, USA) (Zhou et al. 2015). All measurements were performed in the morning between 8 a.m. and 11 a.m. at the beginning drought (90 d), during drought (120 d), and the end drought (120 d), on a fully expanded leaf area and with due phytosanitary measures.

### Analysis of the MDA Level

Lipid peroxidation was estimated by measuring the malondialdehyde (MDA) levels at the beginning drought (90 d), during drought (120 d), and the end drought (150 d) (Wang et al. 2019; Ma et al. 2013). Leaf tissues (0.5 g) in 1.2 mL of 0.1 (w/v) trichloroacetic acid (TCA) were centrifuged at 13,000 g for 20 min. An aliquot of the supernatant (0.3 mL) was mixed with 0.3 mL of 0.5% (w/v) thiobarbituric acid (TBA), incubated at 100 °C for 20 min, quickly cooled, and centrifuged at 10,000 g for 10 min. The A532, A600, and A450 values of the supernatant were then recorded.

### Determination of Antioxidative Enzyme Activities

Antioxidative enzyme activities were measured at the beginning drought (90 d), during drought (120 d), and the end drought (150 d). The activities of the antioxidant enzyme were determined by homogenizing 0.5 g of leaf tissue in 4 mL of 50 mM cold phosphate buffer (pH 7.8) containing 1% polyvinylpolypyrrolidone, 1 mM ascorbic acid, and 10% glycerol in a chilled pestle and mortar kept in an ice bath. The homogenate was centrifuged at 13,000 g for 15 min, and the supernatant was assayed. The Superoxide dismutase (SOD) activity was measured as described by Kumar et al. (2008). The peroxidase (POD) and catalase (CAT) activities were determined as described by Wang et al. (Wang et al. 2019).

### DNA Extraction

Plant tissues were fully ground into powder in a mortar with liquid nitrogen. Then DNA was extracted from rice samples and rhizosphere soil using PowerSoil DNA isolation kit (Qiagen, Germany) according to the manufacturer’s instructions. DNA was quantified with a Qubit Fluorometer by using a Qubit dsDNA BR assay kit (Invitrogen, USA), and the quality was checked by running an aliquot on a 1% agarose gel.

### 16S rRNA Library Construction

We performed 16S rRNA gene amplification for archaea and bacteria. Barcoded primers targeting the variable V4 regions of the 16S rRNA genes were used for amplification by the universal primer pairs, 515F (GGACTACNVGGGTWTCTAAT) and 806R (GGACTACHVGGGTWTCTAAT) (Parada et al. 2016; Walters et al. 2016; Caporaso et al. 2011). Both forward and reverse primers were tagged with Illumina adapter, pad, and linker sequences. PCR enrichment was performed in a 50 μL reaction containing 30 ng template, 2 μL fusion PCR primer (10 μM final concentration), and the PCR master mix (Promega). The PCR cycling conditions were as follows: 95 °C for 3 min, 30 cycles of 95 °C for 45 s, 56 °C for 45 s, 72 °C for 45 s, and final extension for 10 min at 72 °C for 10 min. The PCR products were purified using Agencourt AMPure XP beads and eluted in the elution buffer. The libraries were qualified by the Agilent Technologies 2100 bioanalyzer. The validated libraries were used for sequencing on the Illumina HiSeq 2500 platform (BGI Genomics, Shenzhen, China) following the standard Illumina pipelines, and 2 × 250 bp paired-end reads were generated.

### Bioinformatics Processing and Statistical Analysis

Amplicon sequences were analyzed using the QIIME2 (version 2019.7, heeps://qiime2.org) (Bolyen et al. 2019). We employed the QIIME2-DADA2 plugin to denoise the sequences. All amplicon sequence variants (ASVs) identified as belonging to chloroplasts and mitochondria were removed from the data set. Sequences were classified taxonomically using the Greengenes 13.8 database (McDonald et al. 2012). The potential microbial phenotypes were predicted with Bugbase with Greengenes 13.5 database (Ward et al. 2017; Louca et al. 2016). Statistical analyses of the 16S rRNA microbiome sequencing data, such as Kruskal–Wallis, UniFrac, and PERMANOVA, were conducted in the QIIME2 environment and R version 3.5.1 (Caporaso et al. 2010; Bolyen et al. 2019; Team 2014). The co-occurrence networks were inferred based on the Spearman correlation matrix constructed with R using the 'igraph' package. To meet assumptions of homogeneity of variance, data were log-transformed when required.

To evaluate the phylogenetic community composition, the phylogenetic diversity (PD) and the standardized effect size of the mean pairwise distance (MPD) vs. null communities (mntd.obs.z, equivalent to -NTI (the nearest taxon index)) were calculated for each sampling plot, and the β nearest taxon index (βNTI) was calculated for paired joined plots. All the mean nearest taxon distance (MNTD) analyses were calculated in the R 'picante' package. Phylogenetic diversity (PD) was also were calculated in QIIME2. For both metrics, values between − 2 and + 2 values indicate the expectation under neutral community assembly while the individual values below − 2 or above + 2 are statistically significant (Fan et al. 2018). To confirm whether the niche or neutral processes determined the microbial structure within a sample, pre-emption, broken stick, log-normal, and Zipf–Mandlebrot models (Omelcuk et al. 1977; Macarthur 1957; McGill et al. 2007) were selected to identify the rank species abundance distributions and were calculated in the 'vegan' package in R (Team 2018; Oksanen et al. 2014). All models were compared based on the Akaike information criterion (AIC), which measures the relative quality of a statistical model (Shi et al. 2018).

The null-model-based β-diversity metric (β_RC_) (Chase et al. 2011)was used to evaluate the differences in species richness by modifying the Raup-Crick measure (Zhou and Ning 2017). β_RC_ can be estimated for each pair of communities based on taxonomic cooccurrence data. If the value of Bray–Curtis-based Raup-Crick index (RC_Brey_) is > 0.95 (alpha = 0.05 by a two-tailed test), the given pair of communities share significantly fewer species. If the RC_Brey_ value is less than 0.95, the given pair of communities share significantly more species than expected by random chance. The community assembly mechanism was calculated using phylogenetic bin-based null model analysis (iCAMP) (Ning et al. 2020). Niche breadth was calculated using Levins’ niche breadth index (B) equation, ${\text{B}}_{{\text{j}}} = \, 1/\sum\nolimits_{{{\text{i}} = 1}}^{{\text{N}}} {{\text{P}}^{2}_{{{\text{ij}}}} }$ (Xiong et al. 2020). The B value representing the community level (Bcom) was calculated as the average of B values from all taxa occurring in one community.

We combined the representative ASV sequences from our dataset and aligned them with MAFFT (Katoh et al. 2002). The phylogenetic tree was constructed with IQTree (Minh et al. 2020), and displayed with the Interactive Tree of Life tool (iTOL) (Letunic and Bork 2016). The phylogenetic signals of binary traits that reflect the environmental preferences of various taxa were measured by the D-test of Fritz and Purvis. The phylogenetic dispersion (D) of the drought response (positive or negative) was determined using the 'phylo.D' function in the 'caper' R package (Orme 2013). This phylogenetic dispersion (D) value, developed by Fritz and Purvis, compares the observed sister-clade differences in a trait against those of a random phylogenetic pattern (Fritz and Purvis 2010). Simulated values of D to set points of 0 (as phylogenetically conserved as expected under a Brownian threshold model) and 1 (random). Given a rooted phylogenetic tree and the presences/absences of a binary trait for each tip, the mean phylogenetic depth (τ_D_) at which the trait is conserved across clades is calculated, in terms of the consenTRAIT metric (Martiny et al. 2013; Isobe et al. 2019).

### Accession Number(s)

Raw data and sample metadata are publicly available under the NCBI SRA database with the accession number as No. PRJNA631648.

## Results

### The Effect of the Drought Treatments on *Oryza longistaminata* Performance

To test the plant-associated microbiome response to drought stress, drought was imposed on *Oryza longistaminata* for two months. Two months after water withdrawal, the soil moisture content in the treated samples decreased from 34.6 to 0.72% (Additional file 1: Fig. S1). Notably, the drought-stressed plants remained viable throughout the experiment, which indicated *O. longistaminata’s* strong drought tolerance traits (data not shown).

Drought significantly decreased the leaf photosynthetic rate (Pn) (*p* < 0.01) and the leaf intercellular CO_2_ concentration (Ci) (*p* < 0.01), while increasing the stomatal limitation value (Ls) (*p* < 0.01) (Additional file 2: Fig. S2A). The results suggest that drought decreased the leaf photosynthesis of *O. longistaminata* through stomatal closure. To test the degree of membrane lipid peroxidation caused by drought stress, the MDA content was measured. Drought increased the MDA content (*p* < 0.01), suggesting that drought decreased photosynthesis through metabolic impairment (Additional file 2: Fig. S2B). Drought significantly decreased the antioxidant enzymatic activity levels of SOD and POD and while increased the antioxidant enzymatic activity levels of CAT (*p* < 0.05) (Additional file 2: Fig. S2C). Collectively, these data demonstrate that drought treatments lead to a corresponding increase in plant stress.

### Drought Shapes the Microbiome Taxonomic Community Structure in the Different Compartments

**Fig. 1:**
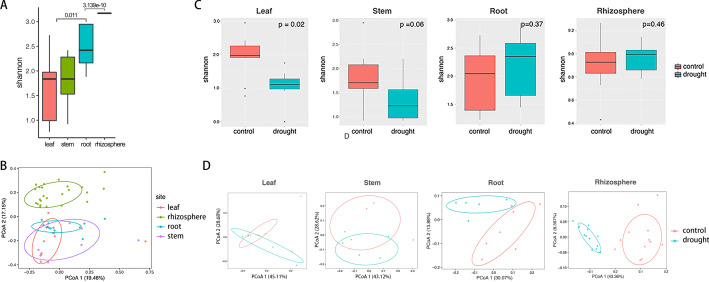
Taxonomic α- and β-diversity estimates. **A** Taxonomic α-diversity indices (Shannon) for the 60 samples from the 4 compartments. **B** Unconstrained principal coordinate analyses (PCoAs) of microbial community composition among the four different compartments of *Oryza longistaminata* at the ASV level based on the weighted UniFrac (WUF). **C** α-Diversity indices (Shannon) of the drought and control treatments in the four compartments. **D** PCoAs of microbial community composition of drought and control treatments among the four different compartments based on the weighted UniFrac (WUF)

A total of 16,188 features and 4,756,919 frequency was obtained among 66 samples with a mean frequency per sample of 72,119 (range 57,060–88,580). The low-quality features were discarded, and plant mitochondria and chloroplast were filtered, resulting in a total of 15,037 bacterial and archaeal ASVs. Alpha rarefaction curves suggested that the richness of the samples has been fully sequenced (Additional file 3: Fig. S3). Across plant compartments, the composition of the microbial communities varied significantly. The taxonomic α-diversity increased from the leaf towards the rhizosphere (Kruskal–Wallis test, *p* < 0.01) (Fig. 1A), the results indicated that the roots and rhizospheres microbiome featured high α-diversity than the leaves and stems. Unconstrained principal coordinate analyses (PCoAs) based on weighted UniFrac distances indicated that the *O. longistaminata* microbiomes within the rhizosphere were separated with other compartments (pairwise.adonis, *p* < 0.01) (Fig. 1B, Additional file 8: Table S2).

The microbial taxonomic α-diversity within each sample was analyzed based on the Shannon diversity index. The mean α-diversity is reduced under drought-stress in the leaves and stems (Fig. 1C). However, the α-diversity of the root and rhizosphere microbiomes did not differ significantly in response to drought (Kruskal–Wallis test, *p* > 0.05) (Fig. 1C). The results indicated that drought reduced the microbial α-diversity in the aboveground compartments (leaves and stems), but not the belowground compartments (roots and rhizospheres).

PCoAs showed that the microbiome was separated by drought in the leaves, stems, roots, and rhizosphere (PERMANOVA, *p* < 0.05) (Fig. 1D). Our data indicated significant compositional shifts between the drought and control group (PERMANOVA, *p* < 0.05).

### Taxonomic Distribution Patterns of Drought-Responsive Taxa

**Fig. 2:**
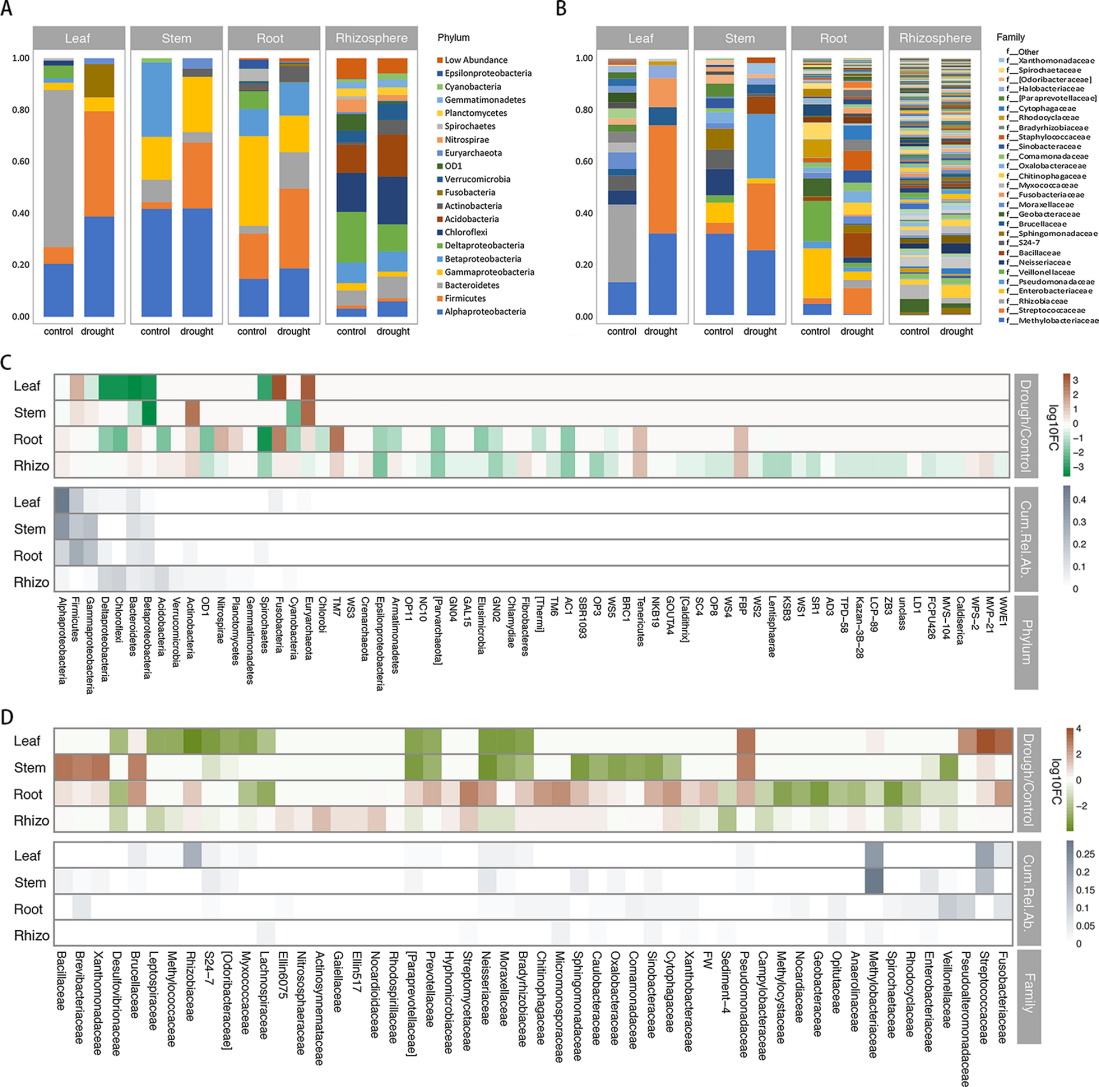
Drought affected the relative abundances in the microbiome. The relative abundances of the most abundant **A** phyla and **B** families in each compartment and drought treatment. Drought-responsive **C** phyla and **D** families in each compartment. The color of the cell indicates the log_10_ fold change in relative abundance concerning the control treatment: an increase appears browner, while a decrease appears greener. The taxa are ranked by their cumulative relative abundance in the whole data set as indicated by the greyscale at the bottom of the plot

We analyze the drought-mediated alterations within the individual compartments. Drought dramatically affected the relative abundances of the microbiome in the leaf, stem, root and rhizosphere (Fig. 2A, B). To more clearly show the patterns of the drought-responsive taxa, we display the top 50 different taxa at the phylum and family levels (Fig. 2C, D). At the phylum level, drought not only affected the major groups but also the minor groups, indicating a broad exclusionary effect of drought on the relative abundances of the microbiome (Fig. 2C).

Consistently with the pattern of α-diversity, in response to drought, the trending patterns of control over their resident microbiota communities in the four compartments were divided into two patterns, aboveground and belowground patterns (Fig. 2C, D). In the aboveground tissues, phyla Firmicutes (*p* < 0.05), and family Streptococcaceae (*p* < 0.05), Fusobacteriaceae (*p* < 0.05), Brucellaceae (*p* < 0.05) were significantly enriched, and phyla Beta-proteobacteria (*p* < 0.1), family Rhizobiaceae (*p* < 0.05) were significantly depleted under drought stress. In belowground compartments, phyla Actinobacteria (*p* < 0.05), TM7 (*p* < 0.1), Bacteroidetes (*p* < 0.1), the family Bradyrhizobiaceae (*p* < 0.001), Chitinophagaceae (*p* < 0.001), Rhizobiaceae (*p* < 0.05) were significantly enriched, and phyla Spirochaetes (*p* < 0.001), Delta-proteobacteria (*p* < 0.05) were significantly depleted under drought stress, and family Desulfovibrionaceae (*p* < 0.001), Geobacteraceae (*p* < 0.05), Rhodocyclaceae (*p* < 0.05), Spirochaetaceae (*p* < 0.05), were significantly depleted under drought stress (Fig. 2C, D, Additional file 9: Table S3, Additional file 10: Table S4). The results indicated that aboveground and belowground plant parts host-microbiome assemblies with different taxonomical structures in response to drought.

### Phylogenetic Distribution Patterns of Drought-Responsive Taxa

**Fig. 3:**
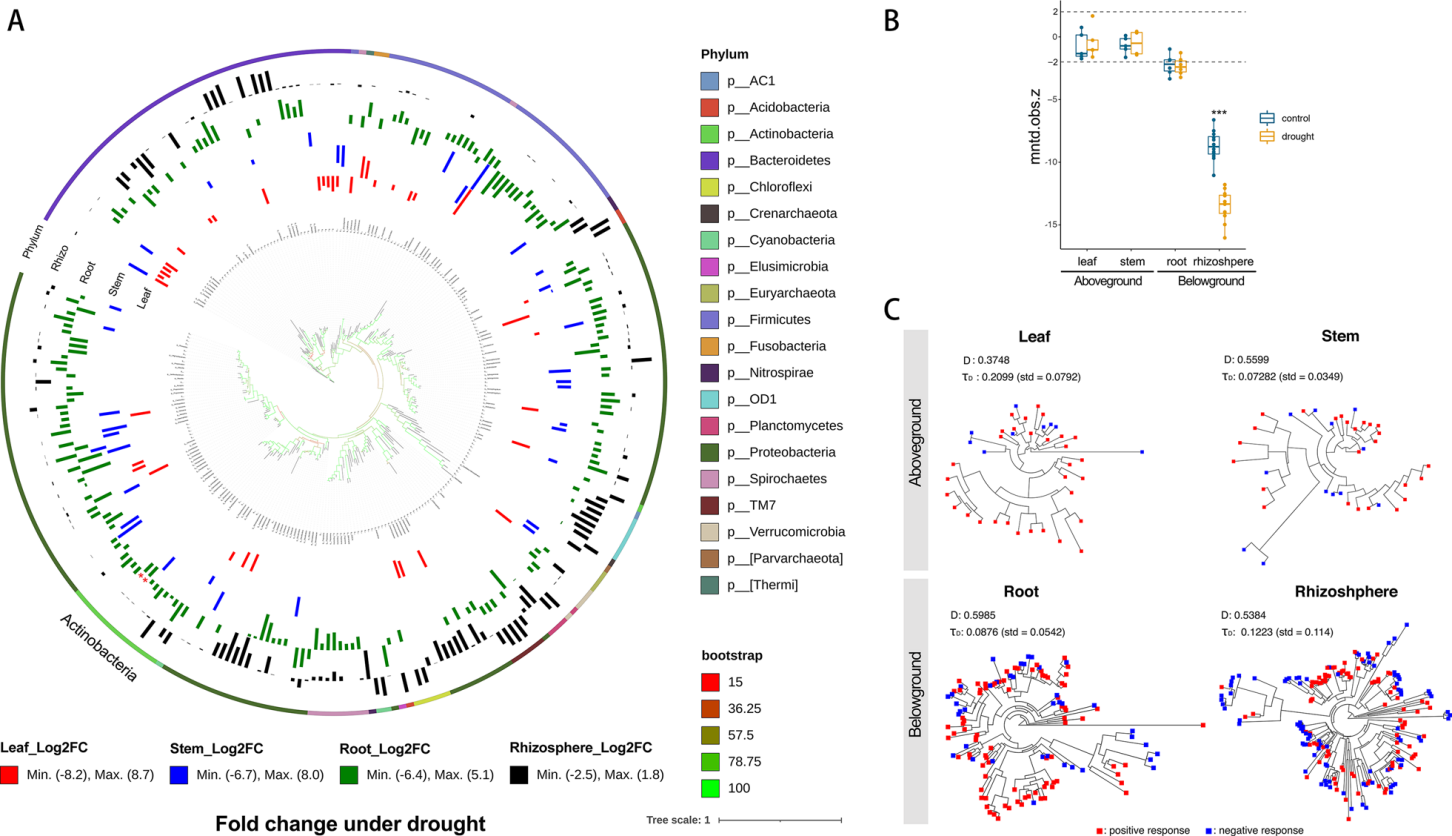
Phylogenetic distribution patterns. **A** Phylogenetic tree of all drought-enriched and -depleted ASVs. The phylogenetic tree at the center of the figure was constructed from one representative ASV sequence from all ASVs with 1.2-log_2_ fold changes (|log_2_FC|> 1.2) in leaves, stems, roots, and rhizosphere of *Oryza longistaminata*. The outer ring colored ring represents the phylum each ASVs belongs to. The middle ring of colored bars represents the relative log_2_-fold enrichment (out) or depletion (in) of each ASVs within drought-treated compartments compared with control compartments. **B** Variation in the standardized effect sizes of the MNTD (SES_MNTD_) of microbial communities between drought and control conditions. Horizontal dashed lines indicate the upper (+ 2) and lower (− 2) significance thresholds. **C** Phylogenetic signal showing the level of trait conservatism under drought. D: Fritz and Purvis index. τ_D_: genetic depth

To identify at higher-resolution taxonomic profiling of the rice microbiome that exhibits relative abundance patterns that differ between control and drought, we investigate change profile under drought for all microbial ASVs using a cutoff of 1.2-log_2_ fold changes (|log_2_FC|> 1.2). A total of 264 ASVs belonging to 86 of the 1064 genera observed within four compartments of *Oryza longistaminat* were identified as having 1.2-log_2_ fold changes abundance between control and drought (Additional file 11: Table S5). A phylogenetic tree constructed from one representative ASV sequence. The results demonstrated that drought-enriched lineages belong almost exclusively to Actinobacteria phyla (Fig. 3A, Additional file 11: Table S5). Two notable exceptions to this pattern (Fig. 3A, asterisks).

To further identify the enrichment pattern in Actinobacteria phyla (Additional file 4: Fig. S4), we investigate the changing profile under drought for all ASVs of Actinobacteria phyla in leaves, stems, roots, and rhizosphere of *Oryza longistaminata*. A phylogenetic tree was constructed, a total of 579 ASVs were identified fold changes abundance between control and drought (Additional file 4: Fig. S4, Additional file 12: Table S6). The results demonstrated that all ASVs were almost exclusively drought-enriched in the belowground compartments, few ASVs were observed in the aboveground compartments. Especially, the enrichment ASVs were found broadly across all the classes of Actinobacteria phyla in the rhizosphere, while all the enrichment ASVs focused on the Actinobacteria class of Actinobacteria phyla in the root.

The microbial community phylogenetic structure of *Oryza longistaminata* in response to drought stress was analyzed. We found that the values of the standardized effect sizes of MNTD (SES_MNTD_, equivalent to -NTI) calculated using the null model were in the range of − 2 to + 2 (Fig. 3B, Additional file 5: Fig. S5, Additional file 13: Table S7) in the aboveground compartments. The values of SES_MNTD_ were <  − 2 in the belowground compartments, suggesting that microbial communities within belowground samples were more significantly phylogenetic clustering among co-occurring species than expected by chance (mntd.obs.z < 0, mntd.obs.*p* < 0.05) (Fig. 3B, Additional file 13: Table S7).

We quantified the strength of the relationship between phylogeny and drought to determine whether phylogenetic information could be predictive of the response of microbial taxa to the global drought stress. The drought responses were strongly phylogenetically conserved in all compartments. The mean genetic depth (τ_D_) ranged from 0.07282 to 0.2099. The D-test of Fritz and Purvis also confirmed that drought responses were dispersed in a mode between Brownian motion and a random model (0 < D < 1) in every compartment, suggesting that closely related species exhibited more similar ecological preferences for drought stress (Fig. 3C).

### The Microbiome Co-occurrence Networks of ASVs Changed with Drought Stress

**Fig. 4:**
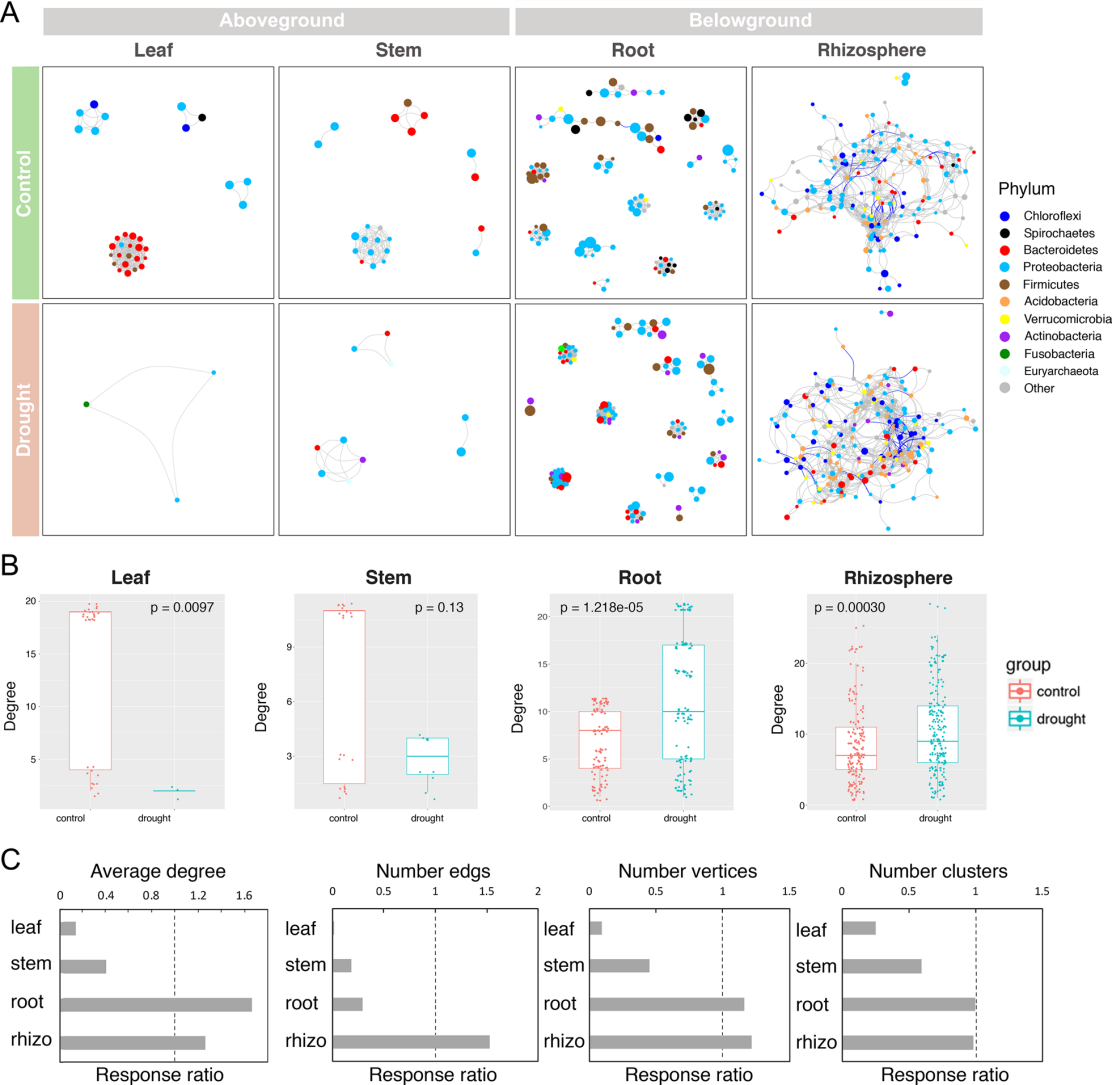
Drought affects the ASV co-occurrence network interactions of the leaves, stems, roots, and rhizosphere of *Oryza longistaminata*. Co-occurrence relationships with strong Spearman’s correlation values (*p* value < 0.5 and abs(r) > 0.6) are depicted for each compartment. The nodes represented a unique ASV in the data sets. **A** ASV co-occurrence network is affected by drought. Networks are colored by phylum and sized based on the relative abundance of the ASV (log10-fold change in relative abundance). The negative correlations of edges are colored blue. **B** Node degree of the co-occurrence network. **C** Response ratios (treatment/control) of the average degree, number of edges, vertices, and clusters within the co-occurrence network

We generated a microbial ASV co-occurrence network using significant correlations to explore the more detailed changes in potential interactions among microbiota under drought stress. Interestingly, we again found that the response of microbial networks to drought was divided into aboveground and underground models. Drought strongly decreased the connectedness of nodes, the number of edges and vertices, and the number of clusters in the aboveground microbial networks, while it increased these properties in the belowground microbial networks (Fig. 4, Additional file 14: Table S8). The data indicated that the belowground microbial networks was more stable than the aboveground networks in response to drought stress.

**Fig. 5:**
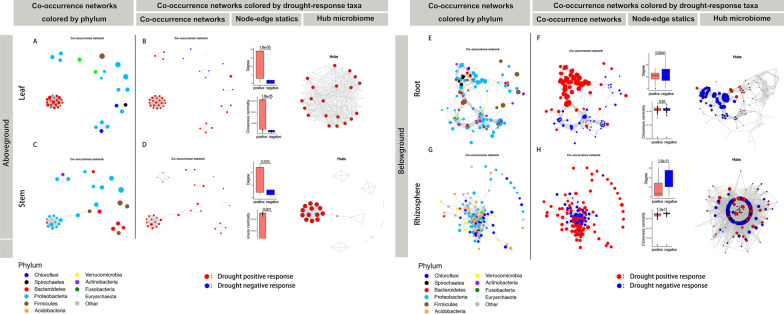
Metacommunity co-occurrence patterns of drought-negative and drought-positive response microbiomes of *O. longistaminata*. Co-occurrence relationships with strong Spearman’s correlation values (*p* value < 0.5 and abs(r) > 0.6) are depicted for each compartment. **A**, **C**, **E**, **G** Community co-occurrence networks. Networks are colored by phylum and sized based on the relative abundance of the ASV (log10-fold change in relative abundance). **B**, **D**, **F**, **H** Metacommunity co-occurrence networks of drought-negative and drought-positive response microbiomes (left panels). The networks are colored and sized based on drought-negative and drought-positive response ASVs. The figures are a summary of node-edge statics (middle panels). The network figure on the right is hub (keystone) microbiome species within the co-occurrence network. Networks are colored based on drought-negative and drought-positive response ASVs and sized based on the hub score of the ASV

Next, we inferred a metacommunity co-occurrence network based on correlation relationships. Drought-positive and drought-negative response ASVs formed independent modules in all four compartments (Fig. 5B, D, F, H, left panels). Notably, the drought-positive response ASVs exhibited much closer interconnections in aboveground compartments, while the drought-negative response ASVs exhibited much closer interconnections in belowground compartments. To verify this observation, we examined the degree and closeness centrality of different groups of ASVs (Fig. 5B, D, F, H, middle panels). The values of these topological features were significantly lower (*p* < 0.05) for drought-negative response ASVs than drought-positive response ASVs in aboveground compartments. While the values of these topological features were significantly higher (*p* < 0.05) for drought-negative response ASVs than drought-positive response ASVs in belowground compartments. Additionally, we focused on how the network hubs (keystone) respond to drought-response ASVs (Fig. 5B, D, F, H, right panels). In the aboveground compartments, drought-positive response ASVs were the dominant network hubs. While, in the belowground compartments, drought-negative response ASVs were the dominant network hubs.

### Microbial Community Assembly Processes

**Fig. 6:**
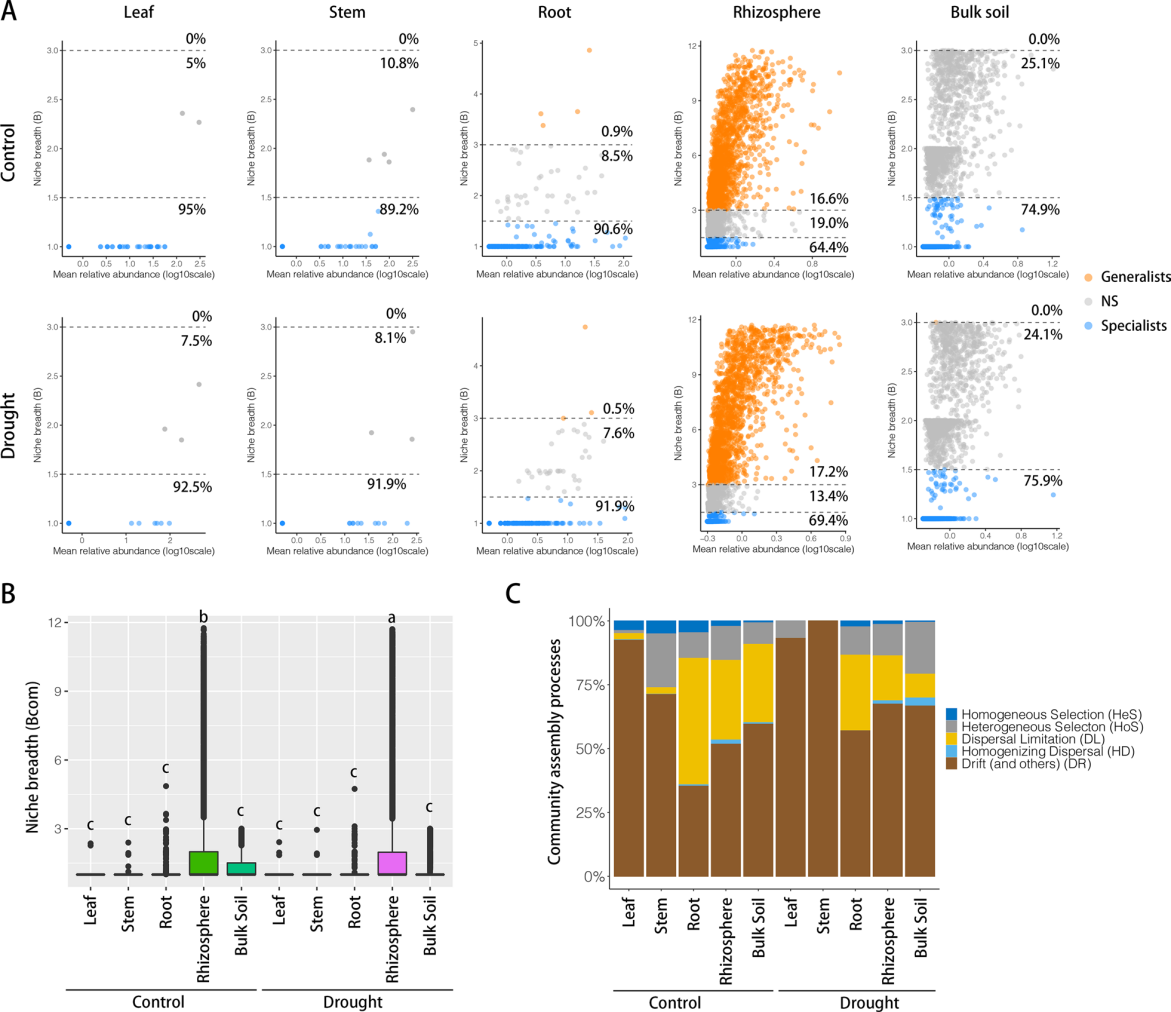
The microbial community assembly processes. **A** The mean abundances of ASVs of microbiome versus habitat niche breadth. The ASVs of temporal generalists are colored orange, the ASVs of specialists are colored blue. **B** Boxplots illustrating mean habitat niche breadth (Bcom). Significant differences (Kruskal–Wallis test, *p* < 0.05) are indicated by different letters. **C** The percentage of turnover in the leaf, stem, root, rhizosphere and bulk soil microbial communities under control and drought stress, governed by deterministic processes (homogeneous and heterogeneous selection), stochastic processes (dispersal limitation, homogenizing dispersal and drift)

We partitioned the microbiome into temporal generalists and specialists. In the leaf, stem and root, specialists were the absolute dominant species (> 89%), while the rhizosphere microbial community has more generalists than those of other compartments (Fig. 6A). In response to drought, the specialists were enhanced among all compartments, while both generalists and specialists were enhanced at rhizosphere. Consistently, the averaged community niche breadths (Bcom) of the rhizosphere microbial communities were significantly highest among all microbial communities (Kruskal–Wallis test, *p* < 0.05), and the Bcom of rhizosphere microbial community were significantly enhanced in response to drought (Fig. 6B). The results indicated that the rhizosphere microbial communities were more metabolically flexible at the community level than those of other compartments in response to drought.

To reveal the ecological drivers controlling microbial community assembly in response to drought stress, we quantitatively infer community assembly mechanisms by phylogenetic bin-based null model analysis (iCAMP). Application of iCAMP to the microbial communities in response to drought stress revealed dominant roles of stochastic processes (drift) in the aboveground compartments, and stochastic processes (dispersal limitation and drift) in belowground compartments (Fig. 6C, Additional file 15: Table S9). The relative importance of dispersal limitation and drift were 1.2% and 89.2% at aboveground, and 32.0% and 52.9% at belowground compartments, respectively. Interestingly, drought decreased ‘dispersal limitation’, and enhanced ‘drift’ among all compartments. Drought decreased the relative importance of ‘dispersal limitation’ 2.5% and 16.7% at aboveground and belowground compartments, and enhanced the relative importance of ‘drift’ 14.8% and 18.7% at aboveground and belowground compartments, respectively.

## Discussion

The plant-associated microbiome extends the functional repertoire of plants in ways that exceed imagination (Berendsen et al. 2012; Bai et al. 2015; Bakker et al. 2018). Crops greatly rely on their microbiota for nutrients uptake, stress protection, and disease resistance (de Vries et al. 2018; Wagner et al. 2016). Deciphering the microbiota-rice interactions under drought, particularly those in drought-tolerant rice, offers great potential for increasing the resilience of rice production to abiotic stress. This study provides a detailed characterization of the survival strategy and microbiome assembly mechanism under drought stress in the above and belowground microbial community of drought-tolerant wild rice (*Oryza longistaminata*).

### Distinct Responses Patterns of the Above and Belowground Microbial Communities to Drought Disturbance

Each compartment of rice can be viewed as an island-like "patch" of habitat occupied by microbial assemblages (Costello et al. 2012). Exploring the responses of microbial communities and their resistance to drought disturbances in each compartment is a central issue for better understanding the mechanisms maintaining microbial diversity in crop-ecosystem (Prosser et al. 2007). Plant adapt to drought by manipulating aboveground–belowground feedbacks between plants and soil microbiota (Lau and Lennon 2012). Plant fitness responses to drought were governed by rapid changes in the belowground microbiome (Lau and Lennon 2012). The aboveground tissue is functionally distinct from the belowground tissue (Chen et al. 2020). Our data also showed that the patterns of control over their resident microbial communities in the four compartments could be classified into two patterns, i.e., aboveground (leaves and stems) and belowground (roots or rhizospheres) patterns. Drought reduced taxonomic α-diversity and destabilized co-occurrence network properties in the aboveground community, but not in the belowground community (Figs. 1, 2). Drought promoted the restructuring and strengthening of belowground network links to more strongly interconnect network properties (Fig. 4). Santos-Medellin et al. reported that drought triggers a compartment-specific restructuring of the rice root microbiome, root microbial communities displaying a more pronounced response than rhizosphere microbial communities (Santos-Medellin et al. 2021). Our data indicated that the aboveground microbial communities were less robust under drought than the belowground microbial communities.

*O. longistaminata* has shown a stronger survival ability than cultivated rice (Wambugu et al. 2015; Yang et al. 2010). *O. longistaminata* had greater stomatal conductance under stress and maintained leaf elongation better under stress than most other rice genotypes. Nevertheless, in response to drought, *O. longistaminata* decreased its leaf photosynthetic rate (Pn), leaf intercellular CO_2_ concentration (Ci), and leaf stomatal limitation value (Ls) to maintain its basic metabolic functions (Additional file 2: Fig. S2). At the same time, *O. longistaminata* developed significantly longer roots and more roots to take up nutrients from the rhizosphere (Liu et al. 2004; Comas et al. 2013). This survival strategy led to the further differentiation of the aboveground and belowground functions under drought stress.

The relationship between biodiversity and ecosystem stability has been well investigated for decades, a positive biodiversity–stability relationship is generally accepted (Tilman et al. 2006; Kuhsel and Bluthgen 2015; Bezemer and van der Putten 2007). The microbiome can exhibit remarkable stability in response to environmental disturbances in natural ecosystems, mainly due to the large population size (Jiao et al. 2019). How the mechanisms underpinning the crop microbial taxa to keep community stability under drought disturbances is unclear. Our data confirmed the relationship between biodiversity and ecosystem stability, the belowground community with higher α-diversity featured more stability than the aboveground community (Figs. 1, 2). Currently, it is challenging to characterize the biodiversity-stability relationship in response to environmental disturbances in microbial ecosystems.

A number of potential causes, such as soil heterogeneity, limits nutrient mobility and access, increases soil oxygen, have been hypothesized to induce a strong shift in microbial community composition (Santos-Medellin et al. 2017; Naylor and Coleman-Derr 2017; de Vries et al. 2020). Under drought conditions, Desulfovibrionaceae (*p* < 0.001) and Geobacteraceae (*p* < 0.001) were depleted in belowground tissues (Fig. 2D, Additional file 9: Table S3). Considering that both groups were strictly anaerobic, the oxygen availability might be an environmental factor that discriminates the microbial community under drought conditions. This is well supported by the Bug base's result showing decreased anaerobic microbes in drought treatment (Additional file 6: Fig. S6B).

### Different Assembly Processes of the Aboveground and Belowground-Communities Under Drought Stress

Given the unequal tolerance of the microbiome to drought, we categorized the species into generalists and specialists. The leaf, stem and root microbiome harbored a higher proportion of specialists, while the rhizosphere harbored a higher proportion of generalists (Fig. 6A). In a given microbial community, some microbe exhibited broad environmental adaptations (generalists), while others showed specific and narrow environmental tolerances (specialists) (Xiong et al. 2020). Consistently, the rhizosphere microbial community exhibited significantly widest niche breadths among all microbial communities (Fig. 6B), which confirms the results from other habitats (Hu et al. 2019; Wu et al. 2018). In addition, the Bcom of the rhizosphere microbial community was significantly enhanced in response to drought (Fig. 6B). The microbial group with a wider niche breadth is thought to be more metabolically flexible at the community level. This may be why we observed that the belowground microbial community diversity and networks stability were kept, but not the aboveground microbial community.

Across microbial ecology, the microbial assemblages are formed by the fundamental processes of community ecology: dispersal, local diversification, environmental selection, and ecological drift. There is a limited understanding of the mechanisms that govern the relative influences of stochastic and deterministic processes in crop-microbe systems, (Dini-Andreote et al. 2015; Zhou et al. 2014).

We estimated the relative contributions of each assembly process over the four compartments in response to drought. In general, environmental perturbations have been classified into two categories: increased nutrient inputs (especially from complex carbon substrates) and disturbances. Nutrient input is believed to increase compositional stochasticity. In contrast, it is generally believed that extreme disturbances such as drought often decrease compositional stochasticity (Zhou et al. 2014). Null model analysis revealed that stochastic (neutral) processes act on microbial community assembly in response to drought stress in both aboveground- and belowground-community (Fig. 6). Other studies show similar results, e.g., that fungi community assembly in drought-stressed sorghum is governed by stochastic processes (Gao et al. 2020). It has been suggested that the complex carbon substrates released by roots provide a resource-rich environment that reduces competitive pressures, which increases compositional stochasticity (Dini-Andreote et al. 2015; Badri et al. 2013). Consequently, we hypothesize that drought-tolerant crops (e.g., wild rice and sorghum) employ stochasticity strategies that are probably mediated by the root exudation to adapt to global change.

### The Unique Role of Drought-Negative Responsive Taxa of *O. longistaminata* in Maintaining Belowground Community Stability Under Drought Stress

Revealing the ecological strategies of co-occurring species within an ecological niche under drought stress is crucial for the understanding of microbial ecosystem function. Wild rice (*O. longistaminata*) shows unique agronomical traits, such as drought-tolerant, we aimed to disentangle the fundamental mechanisms of microbiota communities’ stability in wild rice under drought conditions. Different microbial species exhibit different life traits and occupy varied ecological niches (Potapov et al. 2016). Co-occur microbes may share similar ecological characteristics and life-history strategies (Barberan et al. 2012). Here, we found that the drought-responsive taxa in both aboveground and belowground-community exhibited strong phylogenetic signals and formed independent modules in the co-occurrence network (Fig. 5). The result suggested that similar trait microbiomes not only trend to a response similar in response to drought but also display close interaction.

It is well believed that drought-positive response microbiota will play a dominant role under drought stress in natural ecosystems. Besides, we found that both drought-positive and drought-negative response taxa also play important roles in response to drought. The drought-negative response ASVs exhibited much closer interconnections in belowground microbial community co-occurrence networks, which promoting the belowground microbial community stability (Fig. 5).

### Manipulating Microbiomes to Improve Crop Fitness

Given the beneficial services provided to crops by their microbial symbionts, understanding how these plant-associated microbial communities respond to drought conditions could be an important step in the development of microbial strategies to help increase crop drought tolerance. Plant microbiota control many biogeochemical processes (Martiny et al. 2013). Microbial responses to changing environmental conditions appeared to be phylogenetically conserved (Martiny et al. 2015). For example, microbial responses to soil nitrogen addition were phylogenetically conserved at a genetic depth (τ_D_) of 0.018 (Isobe et al. 2019). Microbial responses such as the ability to produce particular extracellular enzymes (τ_D_ < 0.010) and the use of simple carbon compounds for growth (τ_D_ < 0.010) are less phylogenetically conserved (Zimmerman et al. 2013). While complex metabolic pathways such as oxygenic photosynthesis (τ_D_ = 0.101) and aerobic methane oxidation (τ_D_ = 0.046) are more phylogenetically conserved (Martiny et al. 2013). Our data showed that the mean genetic depth (τ_D_) ranged from 0.07282 to 0.2099 in the four compartments of *O. longistaminata*, suggesting a microbial response to drought stress exhibited strong phylogenetic conservation (Fig. 3C). Therefore, a phylogenetic approach may be useful in predicting how microbial communities respond to environmental changes, and ultimately for the alteration of the biodiversity-driven ecosystem functioning.

Many studies have demonstrated that drought can have considerable effects on microbial communities (Jang et al. 2020; de Vries et al. 2018). In terms of recruitment, we found significant changes in the relative abundances of a broad set of bacteria that spanned many prominent phyla and genera in the microbial community. In particular, the phyla Actinobacteria and Fusobacteria were significantly enriched, and Spirochaetes were significantly depleted under drought stress (Fig. 2). Similarly, Santos-Medellín et al. reported a root-associated community in rice in which several OTUs belonging to the phyla Actinobacteria and Chloroflexi were significantly enriched under drought, whereas OTUs from the phylum Acidobacteria and classes Deltaproteobacteria were generally depleted (Santos-Medellin et al. 2017, 2021). Of note, many works focusing on the root bacterial microbiomes of rice (Santos-Medellin et al. 2017), sorghum (Xu et al. 2018), and diverse lineages of plant species (Naylor et al. 2017; Fitzpatrick et al. 2018) under drought stress have reported enrichment of bacteria from the phylum Actinobacteria (Hartman and Tringe 2019). Actinobacteria are gram-positive (G^+^), monoderm bacteria. G^+^ bacteria (Actinobacteria, Chloroflexi, and Firmicutes) are thought to accumulate under drought, and the cell wall is thought to improve cell drought tolerance by increasing their ability to resist desiccation (Hartman and Tringe 2019; Harris 1981; Xu and Coleman-Derr 2019). Our data also show that G^+^ bacteria were significantly enriched in the rhizosphere under drought conditions (Additional file 6: Fig. S6).

The study could improve our understanding of the maintenance of microbial diversity, and facilitate the prediction of microbial responses to global change in agricultural ecosystems, and provide some strategies for improving crop production. Such strategies may include: (1) the discovery and inoculation of plant growth-promoting microbes into agricultural fields, such as the phyla Actinobacteria and Fusobacteria, the genus *Streptomyces*; (2) the manipulation of crop genetic pathways that regulate microbiota homeostasis, such as the genes that regulate roots exuding complex carbon compounds, could lead to a more beneficial and drought-resilient microbiota, which could, in turn, improve the performance of natural ecosystems and crops; (3) the closer interconnections of drought-negative responsive taxa used as an important microbial trait to screen excellent drought-tolerant rice; (4) the management of soil microbiota through agricultural practices that promote plant drought tolerance. We hope that our results will assist in integrating microbiota into the practices and tools used in modern agriculture.

## Conclusions

Manipulating microbiomes to overcome crop drought stress is an emerging strategy in agricultural systems. Understanding the fundamental mechanisms for maintaining the crop microbial community stability under drought disturbances is critical to the manipulation of the crop microbiome. This study demonstrates that drought-tolerant wild rice (*O. longistaminata*) made aboveground–belowground patterns to overcome drought stress, including enrichment of Actinobacteria in belowground compartments, highlights the important role of the stochastic assembly process in governing the microbial community assembly, and points out the important role of drought-negative response taxa in maintaining belowground microbial community stability under drought disturbance, and have implications relevant to drought protection strategies using root-associated microbiota.

## Supplementary Information

**Additional file 1: Fig. S1.** Soil water content. Statistical significance was identified by the Wilcoxon test with false discovery rate (FDR)-corrected pairwise *P* values. *, *P* < 0.05; **, *P* < 0.01; ***, *P* < 0.001.

**Additional file 2: Fig. S2.** Drought-induced physiological response phenotypes of *Oryza longistaminata* over time. (A) Photosynthetic parameters. Pn: net photosynthetic rate, Cs: stomatal conductance, Ci: intercellular CO2 concentration, Tr: transpiration rate, Ls: stomatal limitation value, WUE: water use efficiency. (B) MDA content. (C) Antioxidant enzyme activity. *P < 0.05, **P < 0.01.

**Additional file 3: Fig. S3.** Rarefaction curves of the observed OTUs (A) and Shannon (B) against sampling depth for each sample.

**Additional file 4: Fig. S4.** Phylogenetic tree of all ASVs in *Actinobacteria phyla*. The outer ring colored ring represents the class each ASVs belongs to. The middle ring of colored bars represents the relative enrichment (out) or depletion (in) of each ASVs within drought-treated compartments compared with control compartments.

**Additional file 5: Fig. S5.** Variation in Phylogenetic α-diversity (PD), the standardized effect sizes of the MNTD (SES_MNTD_), and the standardized effect sizes of MPD (SES_MPD_) of microbial communities between drought and control conditions.

**Additional file 6: Fig. S6.** Discrepancies in microbial community phenotypes between the control group and the drought group. BugBase identified phenotypes associated with Gram-positive bacteria (A), anaerobic bacteria (B), facultatively anaerobic bacteria (C), and stress-tolerant microbiota (D). Statistical significance was identified by the Wilcoxon test with a false discovery rate (FDR)-corrected pairwise *P* values. *, *P* < 0.05; **, *P* < 0.01; ***, *P* < 0.001.

**Additional file 7: Table S1.** Soil texture data.

**Additional file 8: Table S2.** Pairwise permanova results.

**Additional file 9: Table S3.** The degree of fold change under drought at the family level.

**Additional file 10: Table S4.** The degree of fold change under drought at the phylum level.

**Additional file 11: Table S5.** The degree of fold change under drought at ASV level.

**Additional file 12: Table S6.** The degree of the fold change of ASV under drought in Actinobacteria phyla.

**Additional file 13: Table S7.** The results of MPD, MNTD, SES_MPD_, and SES_MNTD_.

**Additional file 14: Table S8.** Network property of co-occurrence networks.

**Additional file 15: Table S9.** Microbial community assembly process in each group.

## Data Availability

Raw data and sample metadata are publicly available under the NCBI SRA database with the accession number as No. PRJNA631648.

## Abbreviations

*O. longistaminata*: *Oryza longistaminata*
*O. sativa*: *Oryza sativa*
*ASV*: *Amplicon sequence variants*
Pn: Net photosynthetic rate
Cs: Stomatal conductance
Ci: Intercellular CO_2_ concentration
Tr: Transpiration rate
Ls: Stomatal limitation value
WUE: Water use efficiency
MDA: Malondialdehyde
TCA: Trichloroacetic acid
TBA: Thiobarbituric acid
SOD: Superoxide dismutase
POD: Peroxidase
CAT: Catalase
PD: Phylogenetic diversity
MPD: Mean pairwise distance
NTI: Nearest taxon index
βNTI: β Nearest taxon index
MNTD: Mean nearest taxon distance
PD: Phylogenetic diversity
AIC: Akaike information criterion
β_RC_: The null-model-based β-diversity metric
RC_Brey_: Bray–Curtis-based Raup–Crick index
iTOL: Interactive Tree of Life tool
τ_D_: The mean phylogenetic depth
PCoAs: Unconstrained principal coordinate analyses
SES_MNTD_: The standardized effect sizes of MNTD
DNCITP: The drought-negative response taxa exhibited much closer interconnections than the drought-positive response taxa
G^+^: Gram-positive
