# Supplementary figures and images for: Strategies and Structure Feature of the Aboveground and Belowground Microbial Community Respond to Drought in Wild Rice (Oryza longistaminata)
Source: Rice (N Y). 2021 Sep 8;14:79. doi: 10.1186/s12284-021-00522-8 (PMC8426455; doi:10.1186/s12284-021-00522-8)

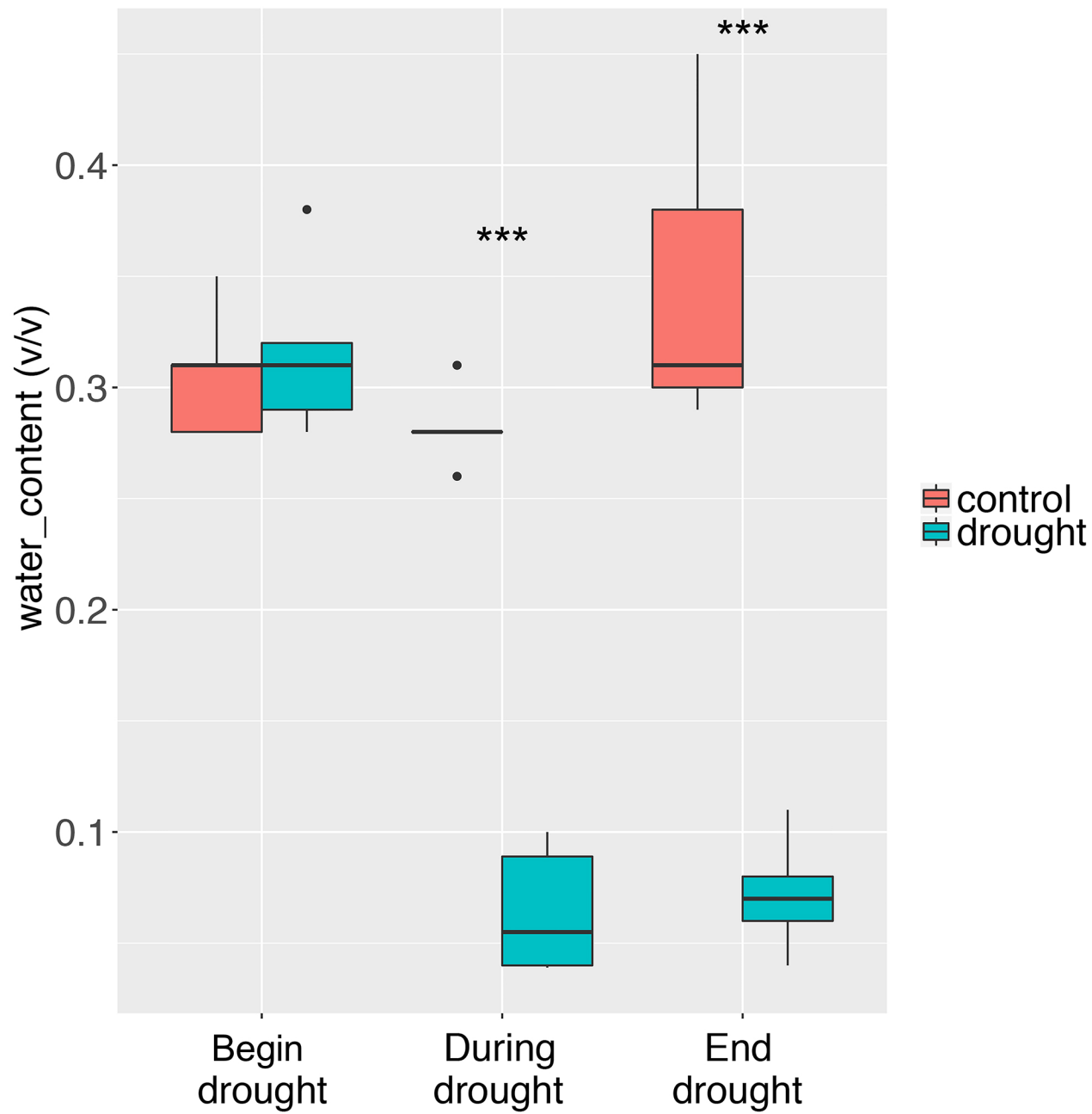

Supplement: Supplementary file 1 — Additional file 1: Fig. S1. Soil water content. Statistical significance was identified by the Wilcoxon test with false discovery rate (FDR)-corrected pairwise P values. *, P < 0.05; **, P < 0.01; ***, P < 0.001. [file 12284_2021_522_MOESM1_ESM.pdf]

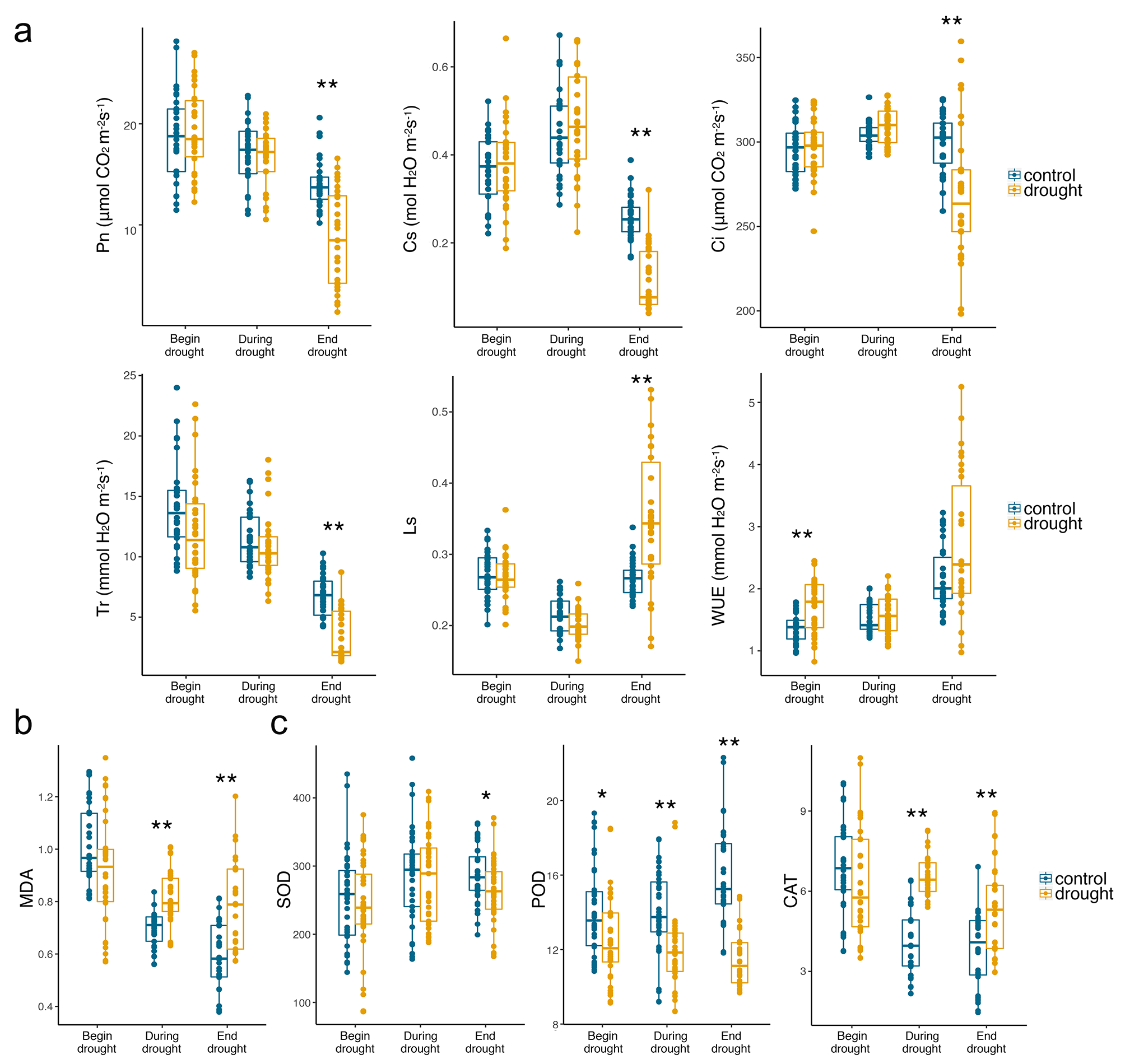

Supplement: Supplementary file 2 — Additional file 2: Fig. S2. Drought-induced physiological response phenotypes of Oryza longistaminata over time. (A) Photosynthetic parameters. Pn: net photosynthetic rate, Cs: stomatal conductance, Ci: intercellular CO2 concentration, Tr: transpiration rate, Ls: stomatal limitation value, WUE: water use efficiency. (B) MDA content. (C) Antioxidant enzyme activity. *P < 0.05, **P < 0.01. [file 12284_2021_522_MOESM2_ESM.tif]

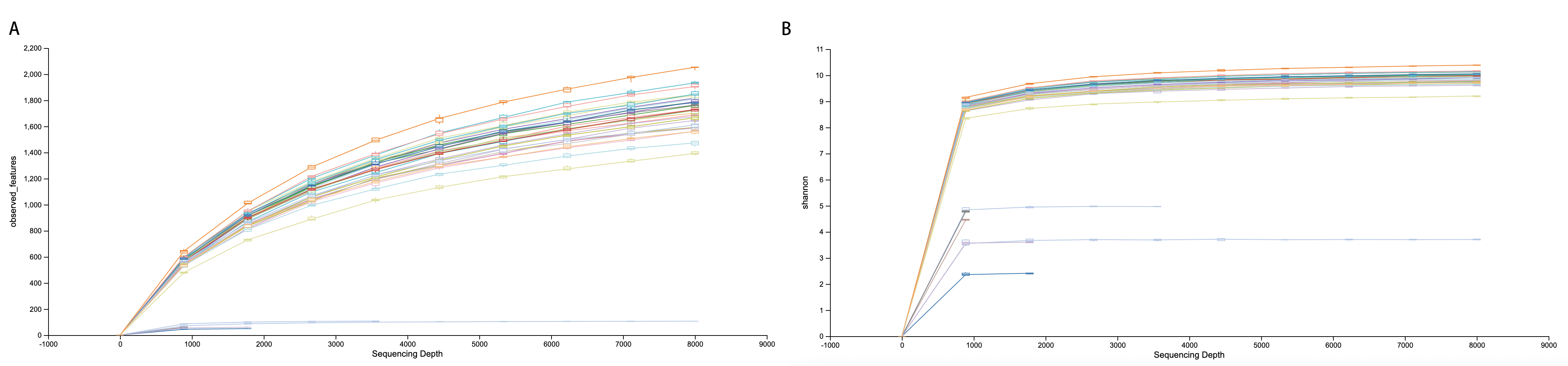

Supplement: Supplementary file 3 — Additional file 3: Fig. S3. Rarefaction curves of the observed OTUs (A) and Shannon (B) against sampling depth for each sample. [file 12284_2021_522_MOESM3_ESM.tif]

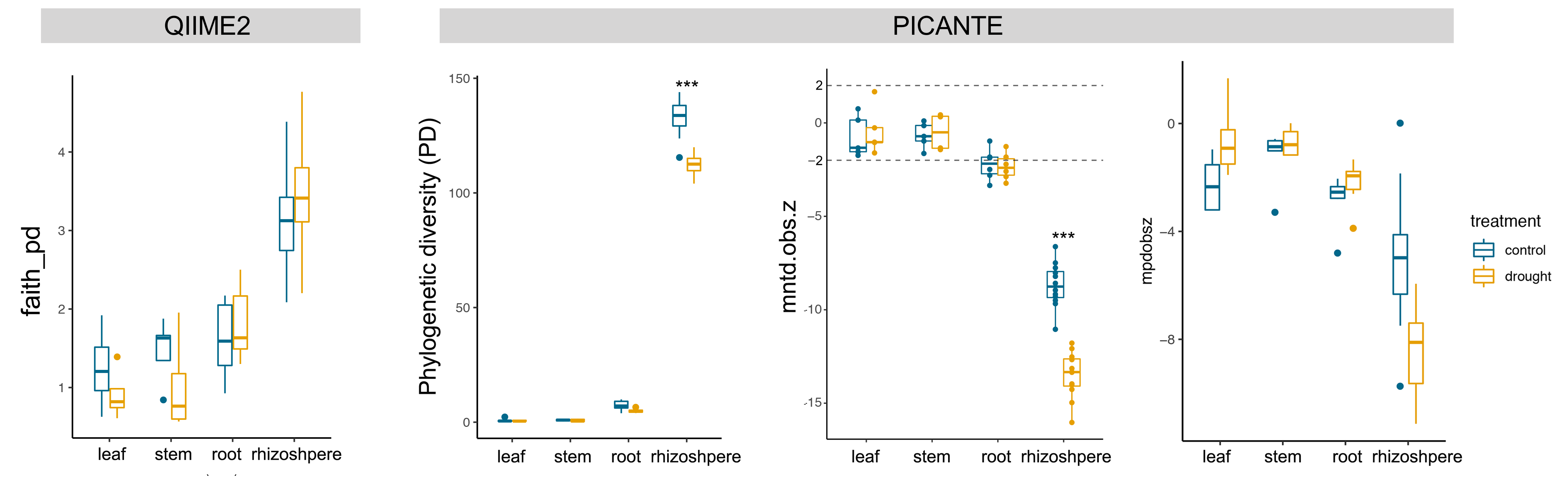

Supplement: Supplementary file 5 — Additional file 5: Fig. S5. Variation in Phylogenetic α-diversity (PD), the standardized effect sizes of the MNTD (SESMNTD), and the standardized effect sizes of MPD (SESMPD) of microbial communities between drought and control conditions. [file 12284_2021_522_MOESM5_ESM.tif]

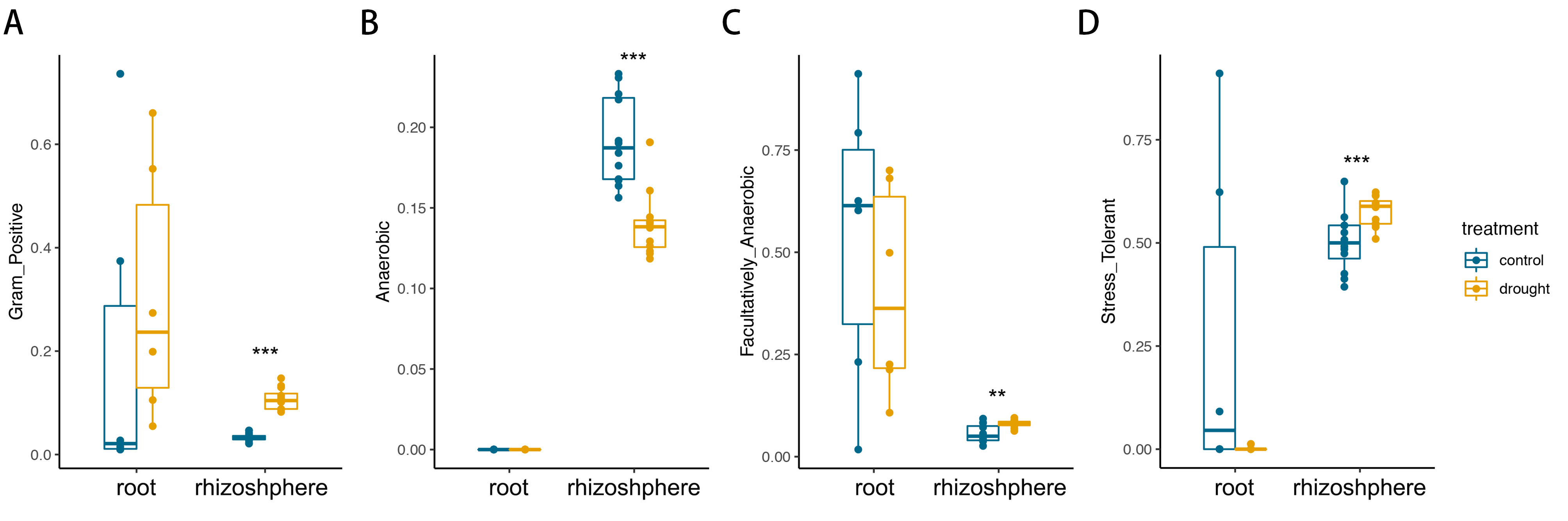

Supplement: Supplementary file 6 — Additional file 6: Fig. S6. Discrepancies in microbial community phenotypes between the control group and the drought group. BugBase identified phenotypes associated with Gram-positive bacteria (A), anaerobic bacteria (B), facultatively anaerobic bacteria (C), and stress-tolerant microbiota (D). Statistical significance was identified by the Wilcoxon test with a false discovery rate (FDR)-corrected pairwise P values. *, P < 0.05; **, P < 0.01; ***, P < 0.001. [file 12284_2021_522_MOESM6_ESM.tif]
